# How Implementation of Cognitive Assistive Technology in Home-Based Services for Young Adults with Intellectual Disabilities Influences Support Staff`s Professional Practice

**DOI:** 10.1177/17446295221083137

**Published:** 2022-04-01

**Authors:** Sylvia Söderström, Hege Bakken, May Østby, Karl E. Ellingsen

**Affiliations:** Faculty of Medicine and Healthscience, Department of Neuromedicine and Movementscience, 87362 Norwegian University of Science and Technology, Trondheim, Norway; 5562 Molde University College, Molde, Norway; Østfold University College, Halden, Norway; Faculty of Medicine and Healthscience, Department of Neuromedicine and Movementscience, 87362 Norwegian University of Science and Technology, Trondheim, Norway

**Keywords:** cognitive assistive technology, implementation, intellectual disabilities, professional practice, support staff

## Abstract

The implementation of technology in home-based services takes place in contextualised socio-material practices negotiated through interactions between a diversity of actors. Prerequisites for succeeding in this process are time and competence, and that use of the technology is experienced as meaningful, for both the support staff and the resident. In this article, we investigate how implementation of cognitive assistive technology (CAT) in home-based services for young adults with intellectual disabilities influences the support staff`s professional practice. The participants are eight support staff members, five women and three men. Data were collected through two group interviews, the second one 8 months after the first one. The data were analysed according to a systematic text condensation (STC) approach. Implementation of CAT is a demanding process, revealing different perspectives on professionalism and changing power relations, and entails a new way of working for the support staff.

## Introduction

In recent years, innovative technologies have been implemented in people’s homes as part of municipalities’ home-based healthcare services. However, it has transpired that in Norway 90% of technology implementation projects have been directed towards elderly people with cognitive challenges, whereas only 5% have been directed towards young people with cognitive or physical disabilities (Knarvik et al., 2017). Moreover, research on the use of technology by people with intellectual disabilities has a brief history in (name of country). To our knowledge, research on how using cognitive assistive technology (CAT) can influence the lives of persons with intellectual disabilities – especially those with moderate and severe intellectual disabilities – has been rare. As a result, current understanding about whether – and, if so, how – use of CAT influences the everyday life of persons with moderate and severe intellectual disabilities remains limited.

Whenever a new technology is implemented as part of a service provision, this decision entails a hypothesis about how this technology shall interact in the service, what role it shall be given and the results that are anticipated. The purpose of using CAT is, for instance, to support thinking, remembering and learning (Janeslätt et al., 2014). Using CAT can also increase independent activity and self-confidence (LoPresti et al, 2004; Østby et al., 2021). Wennberg and Kjellberg (2010) observed that young adults with mild intellectual disability increased their participation in everyday activities, exercised greater control and gained health-related benefits when using CAT in their daily lives versus not using it. Gillespie et al. (2012) found increasing evidence that using CAT supports the individual’s attention, emotion regulation, experience of self and memory.

The implementation of CAT in home-based services takes place in contextualised socio-material practices negotiated through interactions between a diversity of actors. Prerequisites for succeeding in this process are time and competence. It takes time to work through the introduction of the technology, and the use of the technology requires professional competence and social competence. Moreover, it is found to be of vital importance that the implementation of CAT is experienced as including and meaningful for both the support staff and the end user (Michelsen, 2019). In our study, it was an electronic planning device called a Memo Planner (MP) that was implemented into a home-based care service for young adults with moderate to severe intellectual disabilities. An MP is a portable electronic timekeeping and planning device about the size of an iPad that is connected to the internet. Using digital text, pictures and voice, an MP displays the time of activities and the persons involved in those activities on a portable digital screen. In addition to providing an overview and structure of the day and its activities, the inserted text, pictures and voice components may also be used in communication and interpersonal interactions.

Adolfsson et al. (2014) found that implementing new technology had to be individually adapted to each user and that the equipment needed to be easy to manage. Such findings suggest that implementing CAT in home-based services is challenging because it requires a thorough understanding of the individual user’s abilities and daily practices. Investigating the use of electronic planning devices amongst people with cognitive disabilities, Adolfsson et al. (2014) found that members of the support staff needed considerable technological competence. However, a review of the literature on the use of CAT revealed that persons with moderate and severe intellectual developmental disabilities are seldom included in the samples of research on the use of CAT (Gjermestad et al., 2017).

Receiving home-based services involves opening your home to professionals and making your home a workplace for professional practice. Professional practices may be described as practices that are predefined, and professional, theoretical and scientifically based. They entail professional craftsmanship, knowledge and personal insight (Kemmis, 2011). Practices are constructed in relations and interactions between actors and develop in social settings where there are clear frameworks, rules and norms, and a mutual understanding of goals or history (Schatzki, 2012). Studies of professional practices need to include entities outside the individual practitioner who performs the practice. Such entities may be social, discursive, value based, or cultural or historical entities (Kemmis, 2011). Schatzki et al. (2001) point out that all professional practice has two common features: they are *social phenomena* as they include several people, and they are *organised constellations* of different people’s activities. Another common perception is that practices also are founded on unarticulated values. Thus, the practice we observe is just the tip of the iceberg, and what we cannot observe is the unarticulated practice knowledge and discursive practices that justify the practice as moral and ethically acceptable (Gherardi, 2009).

While care-work traditionally has been a family responsibility, it is now a field of public responsibility (Mjøen, 2019). In the field of public home-based care services, encounters take place between what Habermas (1987) calls the *world of systems* and the *lifeworld*. In the world of systems, it is an external perspective that is valid, while in the lifeworld an internal perspective counts. In the latter, personal bonds lead the interaction, such as families and friends. In the system world, relationships are regulated by formal functions and positions, and people can be replaced. In this world, working hours, shifts and day activity plans are important (Mjøen, 2019). In our context, the world of systems is represented through public home-based care services and the support staff working in these services, and the lifeworld is represented by people with intellectual disabilities living in private homes and receiving home-based care services. The contradiction between the world of systems and the lifeworld is not just a practical one but also a crossroad between different logics and needs (ibid.). Despite support staff being of great importance in the process of technology implementation in home-based care for people with intellectual disabilities, little qualitative research exists on what this importance entails.

Thus, this article investigates how a group of support staff at a shared accommodation for people with intellectual disabilities and comprehensive communication challenges experience and perceive how implementation of a CAT, a Memo Planner (MP), impact on their professional roles and practices. We have previously reported on how the residents in this shared accommodation experience this implementation (Söderström, Østby, Bbakken and Ellingsen, 2021).

## Methods

### Design

We chose a qualitative design with an interpretative approach. Such an approach involves investigating people’s social practices and listening to how they talk about those practices, their experiences and their assumptions (Creswell and Peth, 2007). Although human experiences and perceptions are always contextualised and directed towards something, the implicit meaning of ‘something’ in that statement does not have any face value but demands interpretation in order to understand its situational context. Accordingly, our approach was interpretative or hermeneutical, for we, as researchers, interpreted the meaning of participants’ expressed experiences (ibid.).

### Sample

The sample in this study consisted of support staff working at a public shared accommodation for young adults with intellectual disabilities and comprehensive communication challenges. This shared accommodation has separate and private apartments which are the home of the young adults and where they receive home-based care services. At this shared accommodation unit, the electronic planning device – MP – had recently been implemented as part of the care service. Using various software applications, Windows and the internet, the MP can be installed on either the user’s iPad or a large wall-mounted unit.

The support staff received oral and written information about the study. Staff members who wanted to participate in the study gave their consent, and eight staff members gave their consent. This sample consisted of three men (Fred, Guy and Harry) and five women (Ann, Beatrick, Christine, Dorothea and Eva), who were 25–58 years old. The names are fictitious. They had worked closely with the residents during their 2–27 years of work experience in the field. Due to the study`s data collection involving aspects of the residents’ services and everyday life activities, we also obtained consent from the residents` guardians. Residents in the shared accommodation unit and their next of kin or guardians received oral and written information about the study. Seven residents/guardians consented to the study and allowed us to get insight into their use of CAT, their services and everyday activities. These residents, two women and five men from 18 to 40 years of age, had comprehensive intellectual disabilities according to clinical descriptions of low self-determination, living in long-term care and having no or very limited verbal skills (Salvador-Carulla et al., 2011). They also had varying degrees of difficulty with communication and had more or less recently started to use the MP when we commenced the study. For reasons of confidentiality, we had no access to the residents` medical records or other health information. In this article, we do not report on any individual characteristics of the residents. In conducting the study, we followed the guidelines of ethical research by collecting informed consent and ensuring voluntarism and anonymity. The study was approved by the research ethics committee of ….. (Country).

### Data Collection

Due to our perception of professional practices as constructed in relations and interactions between actors, and developing in social settings (Schatzki, 2012), we chose to perform qualitative group interviews with the participants to gain insight into their experiences and perceptions.

#### Group interviews

Group interviews are suitable for investigating the characteristics and dynamics of groups as relevant constitutive forces in the construction of meaning and social practices (Kamberelis and Dimitriadis, 2005). The participants in our study were experienced in their field; they interacted closely with the residents and were in a position to facilitate, promote or prevent new ways of interactions and cooperation by use of the MP. Lasting about 90 min, the first group interview took place with six staff members in an office at their workplace. The second group interview took place 8 months later, involved four staff members in the same office and lasted 75 min. The staff members worked in shifts, with ever shifting staff members at work. Thus, only two of the participants from the first interview were at work when the second interview took place, and two new staff members participated in this interview. In total, eight support staff members participated. The first author conducted and audio-recorded both interviews. Topics discussed in the interviews included the practical use of the MP, how the participants perceived using the MP influenced their workday and practical examples that illustrated their perceptions and potential changes in participant–resident interactions. In this article, we are reporting on how the participants perceived using the MP influenced their workday.

### Analysis

The audio-taped interviews were transcribed verbatim. Then the data were first analysed independently by the three primary authors, who later performed a joint analysis to reach a consensus. Subsequently, the findings were confirmed by the fourth author and discussed in an interdisciplinary research group. The four authors have been trained in conducting research in three fields: special education, sociology and health science. During the analytical process, we aimed to be open-minded, curious and reflective about our preconceptions and to promote the participants’ genuine voices. Although several approaches to analysing qualitative data are available, all share some analytical elements (e.g., the de- and re-contextualisation of data). We analysed the transcripts of the two group interviews as a single data set.

To analyse the transcripts, we used Malterud’s (2012) systematic text condensation (STC) approach. The STC approach consists of four steps: reading all data to form an overall impression, conducting a line-by-line analysis to find meaningful units of text, writing down the condensed meaning of each unit and summarising the content of each condensed unit into themes (Malterud, 2012). The first step provided us with an overall idea of the interviews and the observations. Each of us independently read each interview thoroughly in search of patterns and common features, which enabled us to identify preliminary themes. In the second step, we sorted all text representing the preliminary themes into sub-themes and gave each unit a code. In the third step, we identified meaningful units of text within each code. In the fourth step, we summarised the topic of each coded group, identified the essence of the group and made it a category. During the analysis, five categories emerged: (i) required close collaboration, (ii) tensions became visible, (iii) a demanding process, (iv) changing power relations and (v) a new way of working. Table 1 lists a few examples of parts of our analytical process.

**Table 1.** Examples of parts of the analytical process.

Preliminary topic	Code	Meaningful unit	Category
Things get easier	Someone else says so	‘It is not we who nag’.	Changing power relations
Being coordinated	Doing the same things	‘I think we respond to the residents more alike’.	Required close collaboration
Adaptability	Different prioritizing	‘The technology is important, but we need staff who are experts on ID’.	Tensions became visible
Requirements	Digital intermediary	‘An excellent opportunity to solve the regulatory problem’	Changing power relations
Organizing the work	Routines fall into place	‘Easier to get to work’	A new way of working

## Findings

Even though most of the participants in the study expressed that the implementation of MP in the service was positive and valuable,it turned out that using the MP also brought some challenges. Implementing a new technology like the MP required close collaboration, and through this close collaboration some tensions became visible. While the new practice was experienced as a demanding adaptation, it also led to changing power relations, and a new way of working.

### Required Close Collaboration

The implementation of the MP in the home-care services for the young adults with intellectual developmental disabilities required an even closer collaboration between the support staff members than before the implementation. Being aligned regarding routines, approaches and activities had always been important; however, the implementation of a new technology in the services made alignment even more important. This materialized when it came to how to use the MP. Discussing what made the implementation of the MP successful, Christine expressed her experience in this way: ‘I believe that using the MP makes us more similar in our answers to the residents` many questions. Moreover, it makes us avoid misunderstandings among us if we all use the MP as we are supposed to’. The crucial point here turned out to be ‘if we all use the MP as we are supposed to’. This ‘supposed to’ entailed two important factors: being aligned about how to use the MP and getting equal training in how to use the MP at about the same time. Both factors turned out to be challenging, both organizationally and socially, in a large and diverse staff group who worked in shifts. Nevertheless, several of the participants experienced a close collaboration involving technological use and follow-up as very important. Fred said, ‘The critical success factor is the staff. Because without the staff being punctual and accurate and that everybody follows up, the residents will not get any benefit from using the MP’. Being punctual and accurate referred to the need for every staff member to use the MP in the same way with a particular resident in equal contexts. The follow-ups referred to the need of passing on information to the rest of the support staff about technological shortcomings or errors, or new functions of the MP that were introduced to the resident.

All participants agreed on the need for an even closer collaboration than before between the staff members to make the implementation of the MP successful. They also expressed positive attitudes about using the MP. However, it turned out that the use of the MP also created some challenges and frustrations. Eva expressed it like this:

I think everyone wants to use the MP, and I think everyone sees that using the MP is a good thing. However, you have to be interested. For instance, if the MP does not work, you have to try to solve the problem immediately and to make colleagues aware of the problem. If this is not done, the frustrations increase. This is perhaps where our biggest challenge lies.

Even though everybody seemed to be positive and determined to collaborate closely, some challenges emerged in practice. Some of the staff members were very into technology, some were not interested in technology, and some did not follow-up every technological mismatch or change. This created frustrations in the staff group, frustrations that were challenging to handle.

### Tensions Became Visible

The participants experienced that the emerging and increasing frustrations**in the staff group** illuminated underlying tensions, previously not apparent, but now in the spotlight. Discussing the frustrations, the participants experienced what might be the underlying cause creating the frustrations. As Christine explained: ‘We are a large and complex group of people working here. There is a very big gap in – what should I call it – technology in the precondition and acceptance’. Eva followed up Christine by saying,

*We have been c*hallenged in many ways. We have had intense discussions about technology competence versus professional competence. The tempe*rature has been very high sometimes. I think that must be allowed. You have the ones who want to learn about the technology, and you have a group that wants to learn but does not actually get it. Then you have some that may not want to be involved at all. I feel that we do not have so many of those who don’t want to engage in the technology at all. It is perhaps more that they feel that they have had enough challenges*.

Eva experienced that the staff group was divided, and she categorized this division into three different groups: (i) the ones that wanted to learn, (ii) the ones that wanted, but did not get it, and (iii) the ones that did not want to get involved at all. This indicates a diversity in the staff group but also a tension. Using an MP as part of the home-care services was something the management had decided to implement. The participants expressed the feeling that most of the staff members perceived this as a good thing, improving the service, but that not all staff members shared this perception. According to the participants, some staff members argued that it was more important to use their time and effort in using their professional competence than on technological objects and functions. When the staff members discussed the balance between these two competencies, the participants experienced that the temperature rose. This indicates a difference in perspectives in the staff group. Guy, one of this study`s participants, is a dedicated staff member and interested in technology. He explained what he thought about the staff`s work, their competence and obligation: ‘Primarily, we need to focus on the residents. The technology is of course important, but we need support staff that is competent for the residents’. Initially, it seemed that everybody in the staff backed up the decision to implement the MP, and in theory they did. However, when it came to prioritizing their time and effort in a busy workday, the participants experienced that the staff was divided between the ones who were enthusiastic about the technology, the ones who believed it was beneficial, but struggled putting it to use, and the ones who did not believe it was worth the effort. This might be seen as when unarticulated and taken-for-granted values became articulated it made hidden tensions visible.

### A Demanding Process

Eighth months later, looking back, the participants in the study all thought that implementing the MP had increased the quality of the service and made their workday easier. Nevertheless, they also clearly believed that introducing this technology had been a demanding process. As Fred explained, ‘It has been difficult to get such a large and diverse staff group to understand the intentions and benefits you can get using the technology. It has been a much longer process than I had ever dreamed of’. Harry added:

In the beginning, many colleagues did not see the point in using the MP and all the work it required with implementing it. The purpose of using MP may have drowned in the message that we are a company and that we should do what the management said. Maybe, that is why many thought, “This is not what I want”.

The decision of implementing the MP in the community`s home-based care service was taken by the central management in the community, and this particular shared accommodation unit was chosen as a pilot. Even though the staff members were aware of this and adhered to it, it turned out that in practice it had been more complicated and challenging than anticipated. Ann had some thoughts about why this was the case:

I think that much of the challenging stuff has to do with how we were introduced to the implementation of this new technology. Maybe we should have been included at an earlier stage and received more information about the intention of this implementation.

This illustrates the importance of management being aware of how professional practice develops and consolidates in settings where there is a mutual understanding of frameworks, rules and goals. Beyond learning about the MP being a demanding process, the participants highlighted another change in their work that the implementation of the MP had brought about. This change was experienced as a positive change that made their work less challenging.

### Changing Power Relations

Already from the start, some of the staff members anticipated that the MP could be helpful in some difficult situations they sometimes encountered during a workday. This anticipation motivated them to put extra effort into the implementation process. Eva described her anticipation when she heard about the decision to implement the MP:

When I heard about the possibilities using MP offered, I saw an excellent opportunity to solve the regulatory problem around autistic people. Therefore, I think this is exciting. Very often, it is very difficult to make demands on people with autism.

The participants experienced having to make demands to the residents as difficult, problematic and exhausting. For instance, when a resident should get up in the morning and when he/she should shower was scheduled at the MP. If a resident did not want to get up or shower at the scheduled time the participants found themselves in a situation where they had to make demands about this to the resident. Such demands often resulted in resistance and conflicts. However, implementing the MP had changed something. Instead of the support staff having to tell, or even to command, the resident what to do, they now could point to the MP. As Beatrick explained:

Instead of nagging about what to do we can just refer to the MP. "Look here what’s in here, what should we do here?" Getting things in the system, it is a lot easier, a lot easier to make demands, because now we do not make demands about what to do; the MP makes them.

Dorothea continues:

I feel that I am no longer that bad person who tells the resident what to do. Instead, it is written in the MP that today is hair-washing day. Then it is very rarely a problem when it is not me that decides he has to wash his hair.

For some reason, it seemed that the residents were more willing to do what the technology ‘told’ them to do than what the support staff told them to do. This reduced potential conflicts between the residents and the support staff, something that the support staff experienced as a great relief in their workday. The residents, however, had to do the same things at the same times as before, but now it was the technology that was governing their daily routine. This had led to a shift in the power relations from the support staff being the powerful actor in the relationship to the technology as the powerful actor. At least this is how the participants perceived it, and they appreciated it as it made their workday easier and with fewer conflicts.

### A New Way of Working

After some time, when the support staff got more used to using the MP, the implementation of this technology in the home-based service led to a new way of working for the employees. In many ways, some thought it was a better way. Fred explained: ‘The number of conflict situations between individual residents and support staff has decreased a lot. When the structure and the predictability are in place, it is a lot easier to get to work and do your job’. However, achieving this structure and predictability had not been straightforward for everyone. Looking back, Harry recalled the road they had travelled to becoming accustomed to the MP, saying, ‘Well, there have been some changes and some confusion, and this has been very unfortunate for both the staff and the residents. Therefore, there has been some wear and tear on the staff along the way’. Despite this wear and tear on the staff, it may look like it has paid off in the long run. After almost one year of using the MP, more and more of the staff members use it similarly. Thus, the confusions and tensions are diminished, and the routines seem to fall into place. Ann put words to this development: ‘I think that what has changed is that the staff now acknowledge that this technology has come to stay. The employees are more interested in learning and in implementing this technology’. Things take time to settle, especially changes that are not initiated by oneself or by an explicit common consensus. Nevertheless, the participants agreed that implementing the MP into the home-based service had been an improvement of the service. Fred captured the implementation process by saying: ‘Things are gradually getting better. Even though things are going slow, at least they are going the right way’.

## Discussion

This study found that the implementation of the MP in the home-based service had been a demanding process for the support staff members, revealing different perspectives on professionalism, and changing power relations between the support staff and the residents. Going through this process of implementing an MP in the service had led to a new way of working for the support staff, with closer collaboration between the staff members and a decrease in conflict situations with the residents. An interesting and vital aspect of this implementation process is the issues of coercion and consent from the residents.

### The Process of Implementation

The process of implementation had been a demanding one, requiring closer collaboration between the support staff members than before. Getting everyone in the staff to acquire a mutual understanding and attitude towards using the MP turned out to be a challenge. This may be due to several factors. Even though every staff member was aware of this particular home-based service`s commitment to implementing technological solutions in their service, this commitment was one taken by the central management in the municipality and not by the staff members at the current home-based service unit. The management was quite clear about the importance of the implementation of the technology in the services. Using the MP as a part of the service was a strong expectation, and not really a choice of the individual employee. This top-down decision may have made it hard for staff members who might have been reluctant to accept the implementation to come forward with their concerns. The question is to what degree the employees had other options for work, and how this may influenced their possibility or will to openly criticise any sides of the implementation of technology in the services. Critic can take forms that are indirect, hidden or subtle, such as this study found. Thus, what may seem to be a voluntary consent from the staff members may have an element of self-suppression forced by demands and lack of choices.

Also to be noted are the time and the competence issues. Securing every staff member enough time and adequate training and competence in using the technology is vital for a successful implementation of new technology (Adolfsson, et al., 2014; Michelsen, 2019). Providing everyone with enough information, time and training turned out to be quite demanding in a large and complex staff group that worked in shifts and in various positions. It was especially hard to provide everyone with the same information and the same training, at approximately the same time. Moreover, when things changed or new technological functions were added, it was hard to ensure that everyone was updated and in tune at all times.

How to approach a process of implementation of new technology in a service context with strong traditions and cultures is demanding and important. This is especially true when the implementation affects a small area of service, such as one home-based service unit, within a large organization, such as a municipality, where there is a scarcity of resources and demands are made to use them efficiently. In such a context, it takes will power and a sustained prioritizing by all involved staff members for the implementation to succeed. In this study, it transpired that during the implementation process the organizational and professional challenges were greater than the technological ones. Professional traditions and culture are found to play a significant and crucial role for the outcome of an implementation process in public services (Ellingsen et al., 2019). Thus, it is of great importance to start by anchoring the idea of implementation in the staff group. Lack of competence, uncertainty or attitudes are found to be factors that are important for the implementation of technology (Söderström, 2014). The combination of lack of training in its use and dealing with technology that is generally little used in some workplaces has also been described as a barrier to implementation of new technology in work situations (Bureau of Labor Statistics, 2011). Systematic training and competence building of support staff working with implementation of technology in home-based services is called for and highlighted as a necessary promoting factor for the implementation process (Wigfield et al., 2013).

### Perspectives on Professionalism

In our study, different perspectives on professionalism emerged. Some of the support staff perceived professionalism as competence ‘on the residents’, expressed through face-to-face human interactions, while others believed this competence could just as well be utilized through using technology as a mediator. We also found a varying degree of technology acceptance among the staff members. While some expected the technology to be very helpful in their everyday interactions with the residents and worth the effort of changing their work routines, others found it more bothersome than useful to invest time and effort in the new technology. While this might be understood as technology resistance, it might also be understood as differences in perspectives on professionalism. The initial reluctance to use the MP in their daily practice was due to a scepticism that the technology might endanger the delicate interpersonal, face-to-face interaction that their work relied on.

The perspectives and values that shape the practices of professionals also shape their values (Schatzki et al., 2001). Studying the negotiations and adherence to values in practice, such as listening to the support staff`s experiences and hearing their discussions about the implementation of the MP, revealed that the staff did perceive the implicit value in the technology, as well as how their attitudes and traditions shaped their practice. Values are, however, not constant. Practice constitutes values, and values are transformed into practice. For instance, some of the support staff in our study experienced the implementation of the MP as stressful and uncertain, while others perceived it as bringing increased flexibility and fewer conflicts. Both ways of experiencing implementation of new technology into services are also found in previous studies (Ellingsen and Aas, 2008; Norvoll and Fossestø l, 2010). The value of different practices, whether these are new or old ones, mandatory or voluntary, are shaped by negotiations between mutual professional and individual personal values, and by expressed and inherent values. Our study has highlighted how inherent perceptions and values of professionalism – previously taken for granted – came to the surface and created tensions amongst the members of the support staff. Kemmis (2011) points out that professional practices consist of entities such as values, discourses, tradition and tactical knowledge, just as much as they reflect theory, research and empirical knowledge. Moreover, these entities are often not articulated. Gherardi (2009) describe practices as the activities that make up the tip of the iceberg, and that what lies beneath the surface is a mass of practical knowledge and discursive practices that justify practices as morally acceptable. This ‘underwater iceberg’ that keeps the practice afloat does not necessarily consist of well-considered, reflected and articulated practices (Schatzki, 2012).

### Changing Power Relations by Implementing a Socio-Material Practice

The participants experienced some of the residents’ alternative non-verbal ways of communicating their needs and interests as challenging to interpret. In cases where the staff did not understand the residents` expressions of needs and interests this sometimes lead the residents to alternative ways of expressing needs and of asserting choices, by showing resistance to follow plans and activities scheduled in the MP. This challenge in the two way communication between staff and residentoften led to conflicting situations experienced as challenging and demanding by the support staff. The implementation of the MP led to a decrease in such conflicts, and less use of argumentation and persuasion was needed to carry out the planned activities of the day. This represented a practice that apparently transferred the power of decision from the staff to the technology. Thus, implementing the MP into the service resulted in the institution of a socio-material practice. Such practices are everyday actions and interactions carried out by using various human and non-human resources, such as our bodies, analogue tools or digital technology. A socio-material practice implies that the different resources put to use – whether human or non-human – are all actors mutually reinforcing each other (Latour, 2008; Moser, 2006). The introduction of the MP into the home-based service meant that this technology would become a new actor mediating between the residents and the support staff. The interaction between residents and support staff was supplemented in that some of the interactions were transferred to dialogues between the technology and the resident. This transference decreased potential conflicts between the support staff and the residents. The question now was whether, or how, it changed the power relation between the support staff and the residents. Beyer and Perry (2013) describe how technology can represent a transfer of control from staff to the technological system, which in some cases has the potential to enhance self-determination, while in other cases it can hamper self-determination or make an illusion of self-determination by preventing turmoil. Using CAT, such as an MP is considered a tool for increasing empowerment and self-determination among young adults with mild to moderate intellectual disability (Ramsten et al., 2018). However, staff use of technology is also impacted by the social environment and service arrangements. Svanelö v (2019) concludes in his study of power practices in group homes for people with intellectual disabilities that practices of power are inherent in group homes, and that the residents must conform to, resist or negotiate these practices and position themselves within its hierarchy. We found that even though it appeared that power was transferred from the staff to the residents through the socio-material practice of using an MP, power was actually now just more hidden, as it was the support staff that had inserted which activities the MP should display at a given time. Nevertheless, the staff perceived this change in who decides what to do being transferred from themselves to the technology as a relief making their workday easier and safer.

Any technology is a tool that has multiple possibilities, also including power and control. In our study it is somewhat unclear to which extend the residents had given an informed and free consent to the implementation of the MP a part of their home-based service. Even though all residents had been informed, and had participated in setting up their personal schedule in their MP, with support from their families and parents, it is hard to be sure to what degree the consequences of this was understood by the individual resident. Nevertheless, there were an implicit anticipation among the participants that using the MP was what the residents wanted.

When considering voluntariness and consent versus coercion this is never a question of the outcome of the question at hand. That is, even if the outcome of using the MP helps a resident to perform his/her tasks without having conflicts with the staff, this is not relevant to the questions of consent, voluntarism and coercion. These questions have to be studied on theoretical and ethical premises, supported with information about the person and situation that can give substance to the issue. This applies whether technology is involved or not. What may seem to be a positive interest in technology may well be a need to please, or a desire to let go of something else. Some vital questions are who controls the technology in use, who draw the lines for when to use it or not, who benefits from the use and what is its legitimacy. These questions must be answered in every case as the answers are individually. There is a tendency of looking at technology as a relief on the service provision and a valuable way to allocate scars resources. That is in itself potentially a risk factor for systematically use of coercion and well known in the discourse around use of welfare technology.

However, from the point of view from our participants the implementation of the MP in the home-based-services was experienced as beneficial. Nevertheless it was also as a troublesome and demanding process, revealing tensions among the staff members, while providing a new way of working with less conflicting situations. We found that these experiences also revealed different perspectives on professionalism and apparently changing power relations. We do, however, ask if the power relations were actually changed or just hidden behind technological equipment. In the multidisciplinary field of practice, such as the implementation of CAT in home-based services, further knowledge is needed about what personal and professional competencies and organizational and institutional factors are necessary, and how to successfully facilitate these competencies and factors.
